# Protein Ligation in Living Cells Using Sortase

**DOI:** 10.1111/j.1600-0854.2012.01345.x

**Published:** 2012-03-23

**Authors:** Karin Strijbis, Eric Spooner, Hidde L Ploegh

**Affiliations:** Whitehead Institute for Biomedical Research9 Cambridge Center, Cambridge, MA 02142, USA

**Keywords:** circular polypeptides, intracellular protein engineering, *S. pyogenes* SrtA, site-specific labeling

## Abstract

Sortagging is a versatile method for site-specific modification of proteins as applied to a variety of in vitro reactions. Here, we explore possibilities of adapting the sortase method for use in living cells. For intracellular sortagging, we employ the Ca^2+^-independent sortase A transpeptidase (SrtA) from *Streptococcus pyogenes*. Substrate proteins were equipped with the C-terminal sortase-recognition motif (LPXTG); we used proteins with an N-terminal (oligo)glycine as nucleophiles. We show that sortase-dependent protein ligation can be achieved in *Saccharomyces* *cerevisiae* and in mammalian HEK293T cells, both in the cytosol and in the lumen of the endoplasmic reticulum (ER). ER luminal sortagging enables secretion of the reaction products, among which circular polypeptides. Protein ligation of substrate and nucleophile occurs within 30 min of translation. The versatility of the method is shown by protein ligation of multiple substrates with green fluorescent protein-based nucleophiles in different intracellular compartments.

Sortase A transpeptidase (SrtA) is an enzyme of Gram-positive bacterial origin involved in covalent attachment of proteins to the bacterial cell wall. The enzyme has also been used in a number of protein-engineering applications (reviewed in 1). Sortases recognize substrate proteins bearing a sortase motif, LPXTG for *Staphylococcus aureus* SrtA and LPXTG/LPXTAA for *Streptococcus pyogenes* SrtA, but sortases from other Gram-positive bacteria have yet again different recognition motifs (2, 3) and substrate requirements. After recognition of the sortase motif by SrtA, the catalytic cysteine residue in the enzyme's active site serves as a nucleophile to cleave the peptide bond between threonine and glycine (or alanine). Cleavage occurs with concomitant formation of a thioacyl intermediate between substrate and enzyme. This intermediate is then resolved by the N-terminus of an (oligo)glycine nucleophile, thereby creating a new peptide bond that links the substrate to the incoming nucleophile (Figure 1). We refer to this method of protein labeling as sortagging. The method in principle allows protein modifications at the C-terminus, the N-terminus or both (reviewed in 1).

**Figure 1:**
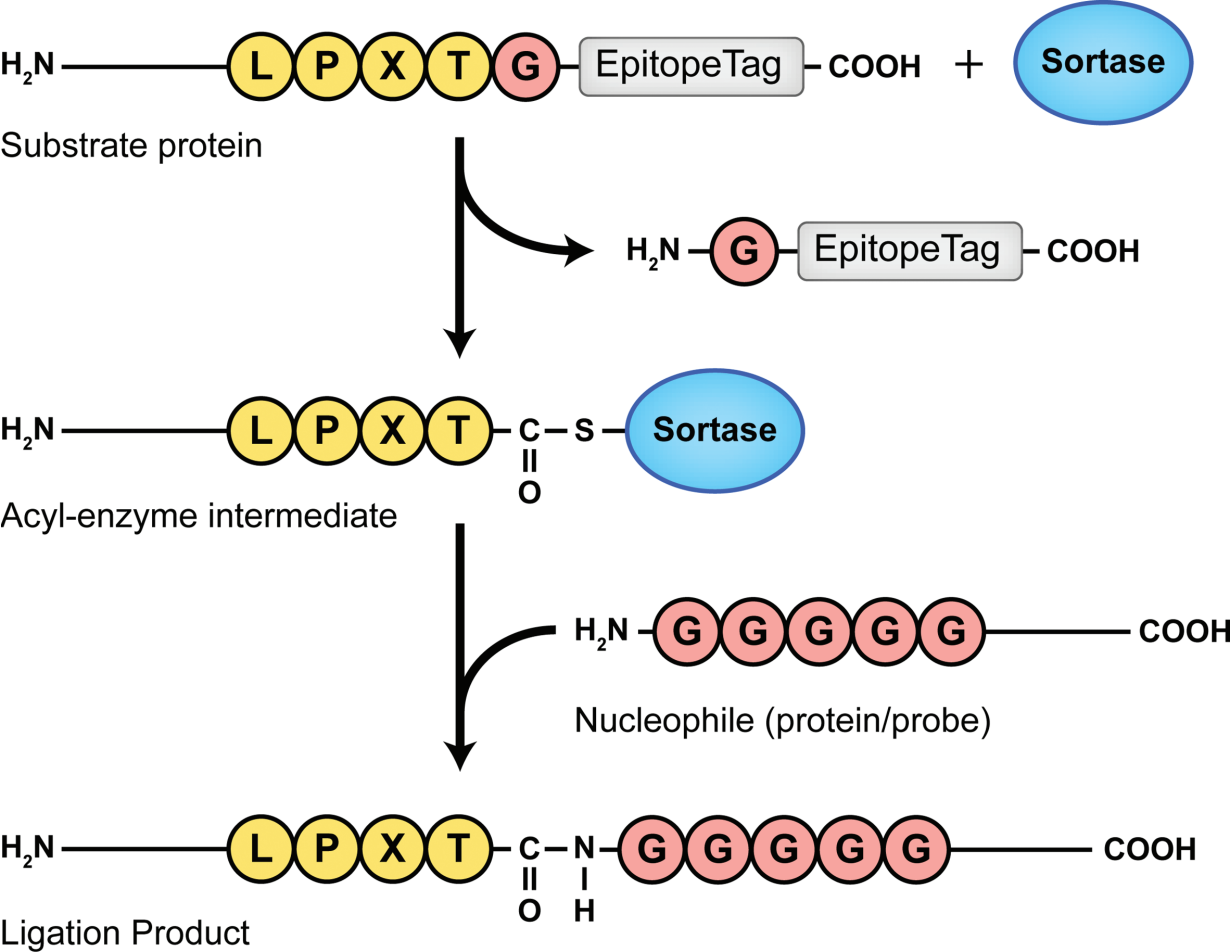
Schematic representation of sortase-mediated protein ligation A substrate protein containing an LPXTG motif near the C-terminus followed by an epitope tag is cleaved between the threonine and the glycine by sortase, thereby releasing the epitope tag (top). The formed acyl-enzyme intermediate can be resolved by an (oligo)glycine-based nucleophile (middle), resulting in the release of sortase and the formation of a substrate-nucleophile ligation product (bottom). Figure adapted from Dasgupta et al. 2011.

Several areas of application of the sortagging method have emerged, ranging from pure chemistry-based to cell biology and biochemistry. The method has proved versatile especially because the enzyme tolerates a wide variety of substrates in proximity of the cleavage site and in nucleophile modifications 2004, the latter including polyethylene glycol chains 2007, lipids 2008, nucleic acids 2007 and a glycosylphosphatidylinositol anchor analog 2009. Biochemical applications of sortase include the *in vitro* sortase-catalyzed circularization of proteins such as enhanced green fluorescent protein (eGFP), four-helix bundle cytokine interferon α3 (IFNα3) and granulocyte colony-stimulating factor-3 (GCSF-3), human erythropoietin (EPO) and the wound-healing peptide histatin-1. The resulting circular polypeptides proved to be more stable and at times more active than their linear counterparts (10—12). The reaction conditions for sortase-mediated transacylation are compatible with the environment of live cells. Membrane proteins exposed at the cell surface can serve as substrates for both C- and N-terminal labeling (13—17), even in complete tissue culture medium supplemented with serum components. The site-specific attachment of the nucleophile, the small sizes of nucleophiles that serve as substrates, the reversibility of the reaction and the mild reaction conditions are all attractive features of this labeling method.

The mild reaction conditions for the sortase reaction, as well as the ability to execute ligation reactions on the surface of living cells suggested that it might be possible to apply sortase to intracellular protein ligation. We thus set out to adapt the sortagging method for intracellular use. We show that the Ca^2+^-dependent *S. aureus* enzyme is not functional intracellularly, but the Ca^2+^-independent *S. pyogenes* SrtA is functional in the cytosol and endoplasmic reticulum (ER) lumen of both *Saccharomyces cerevisiae* and mammalian HEK293T cells. Circularization of proteins in the ER lumen results in secretion of the circular product, opening up the possibility of having recombinant proteins with protein modifications specific for eukaryotes (e.g. N-linked glycosylation) produced and secreted directly as circular products. We also find that GFP-based nucleophiles can be used to sortag multiple protein substrates, both in the cytosol and in the ER.

## Results

### Sortase-mediated protein circularization in the cytosol

A GFP construct with a C-terminal LPETG motif and an N-terminal short stretch of glycines that can function as the nucleophile (G_5_-eGFP-LPETG) can be circularized by *S. aureus* SrtA, resulting in a faster migrating circular GFP species 2009. To address whether bacterial sortases are active in the cytosol of *S. cerevisiae*, we expressed a G-GFP-LPETG-Myc circularization substrate under control of the constitutive CIT1 promoter and co-expressed *S. aureus* or *S. pyogenes* SrtA under control of the inducible GAL promoter. The N-terminal glycine of the G-eGFP-LPETG-Myc construct will be exposed after removal of the N-terminal methionine by methionine aminopeptidase. Induction of sortase expression was performed by shifting the cells from low glucose media to rich galactose media and samples were taken after 0, 4 and 8 h.

**Figure 2:**
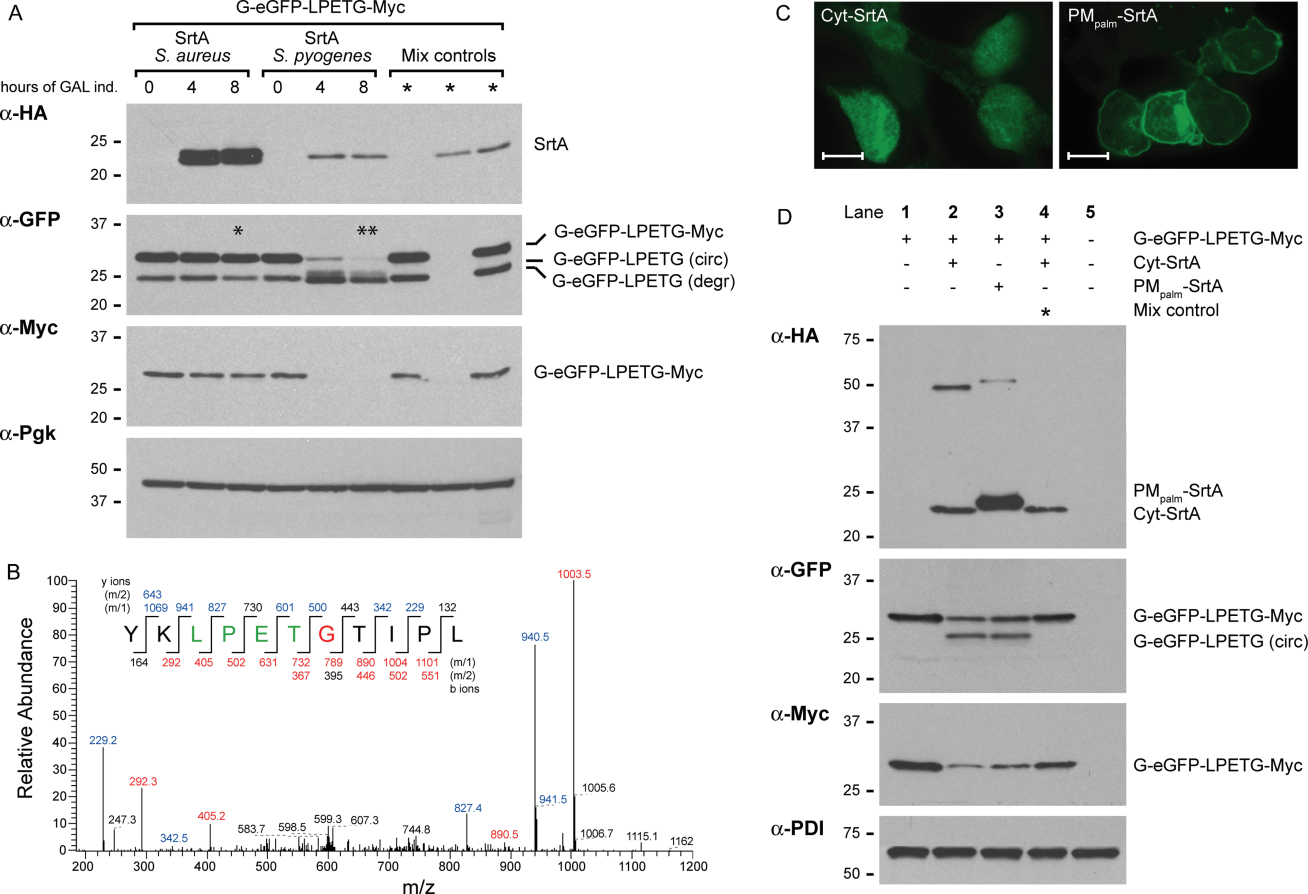
GFP circularization by *S. pyogenes* SrtA in *S. cerevisiae* and HEK293T cytosol A) Galactose induction of *S. cerevisiae* strains expressing cytosolic circularizable G-eGFP-LPETG-Myc substrate under control of the constitutive *CIT1* promoter with *S. aureus* or *S. pyogenes* sortase under control of the inducible *GAL* promoter. Samples were taken after 0, 4 and 8 h of galactose induction. Cells expressing either G-eGFP-LPETG-Myc or *S. pyogenes* sortase (mix control, first two lanes) were mixed before lysis and prepared for immunoblot like the other samples (mix control, third lane). Fractions marked with asterisks were analyzed by MS. B) MS/MS spectrum of a tryptic fragment of the circular GFP showing the ligation of C-terminal residues (YKLPET) to the N-terminal residues (GTIPL). The linkage peptide was found in the (**) sample, but not in the (*) sample. C) Anti-HA immunofluorescence on HEK293T cells expressing *S. pyogenes* sortase targeted to cytosol (Cyt-SrtA) or plasma membrane (PM_palm_-SrtA). Scale bar represents 7 µm. D) Immunoblot analysis of HEK293T cell expressing different combinations of G-eGFP-LPETG-Myc substrate, Cyt-SrtA and PM_palm_-SrtA. Cells expressing either G-eGFP-LPETG-Myc or Cyt-SrtA were mixed before lysis and treated like the other samples (mix control). Experiments were performed multiple times, representative experiment are shown.

*Staphylococcus aureus* SrtA induction did not result in modification of the G-GFP-LPETG-Myc construct (Figure 2A), presumably because the intracellular Ca^2+^ concentration is insufficient for activity of the enzyme, which is strictly Ca^2+^ dependent 2009. However, co-expression of the G-GFP-LPETG-Myc construct with SrtA derived from *S. pyogenes* led to conversion of the input G-GFP-LPETG-Myc polypeptide into a faster migrating GFP species that had lost the Myc tag (Figure 2A). To exclude the possibility that the sortase reaction occurred during or immediately after cell lysis, we mixed cells expressing the G-GFP-LPETG-Myc substrate (mix control, first lane) with cells expressing *S. pyogenes* sortase (mix control, second lane) immediately before lysis (mix control, third lane). This mixing control did not produce the faster migrating GFP polypeptide, indicating that the sortase reaction must have occurred inside the cells. Samples from the *S. aureus* and *S. pyogenes* galactose induction were analyzed for the presence of a circular GFP product by mass spectrometry (MS; marked by asterisks). The presence of the YKLPETGTIPL C- to N-terminal fusion peptide was confirmed in a tryptic digest of the *S. pyogenes* sample (**) and after digestion with chymotrypsin (data not shown) but not in the *S. aureus* SrtA sample (*) (Figure 2B). These data firmly establish the ability of the *S. pyogenes* bacterial sortase, expressed in the cytosol of a eukaryotic cell, to catalyze a reaction thus far observed only *in vitro*.

Next, we explored whether intracellular sortagging could also occur in mammalian cells. Constructs were designed to target *S. pyogenes* SrtA to the cytosol (Cyt-SrtA, similar to the *S. pyogenes* SrtA construct used for *S. cerevisiae*) or to the cytoplasmic face of the plasma membrane by addition of the N-terminal palmitoylation motif of Lyn tyrosine kinase (PM_palm_-SrtA). To investigate the subcellular localization of both sortase variants, we transfected HEK293T cells with the constructs and located sortase by immunofluorescence with an anti-HA antibody. The majority of the Cyt-SrtA construct localized to the cytosol, with some nuclear staining as well, while the PM_palm_-SrtA clearly localized to the plasma membrane (Figure 2C). To investigate the sortagging efficiency of both SrtA variants, we co-transfected HEK293T cells with the G-GFP-LPETG-Myc substrate and Cyt-SrtA or PM_palm_-SrtA constructs. Both variants showed comparable efficiency: for both conditions we observed ∼50% conversion of linear G-GFP-LPETG-Myc into a single, faster migrating GFP band that was absent from the mix control (Figure 2D). The acyl-enzyme intermediates formed between substrate and enzyme were clearly detectable with both anti-HA and anti-GFP antibodies in the HEK293T experiment, while this was not the case for the *S. cerevisiae* experiment.

### Secretion of circular proteins through ER sortagging

**Figure 3:**
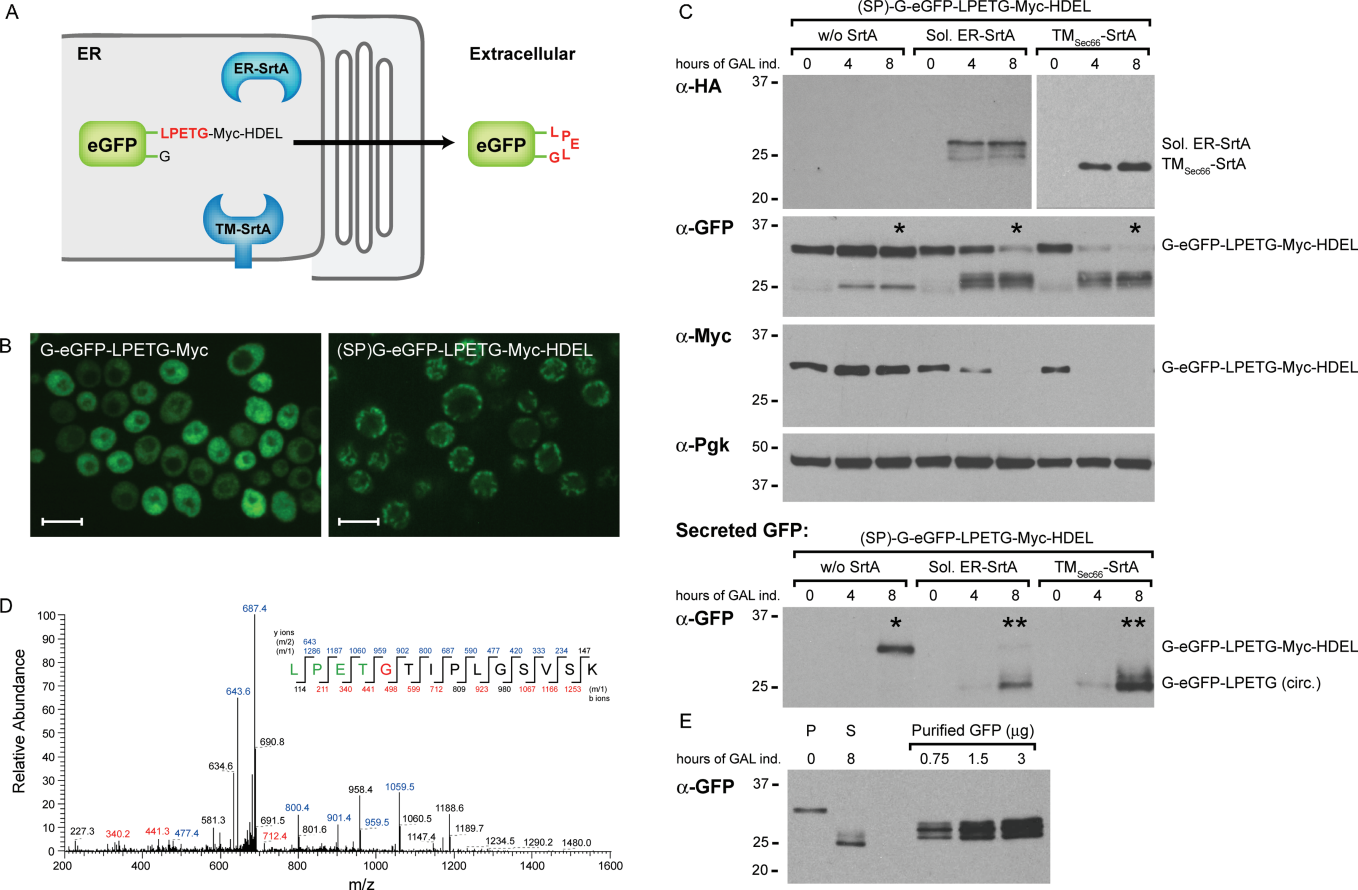
Secretion of circular proteins through ER sortagging A) Scheme showing sortase-mediated secretion of circularized eGFP from the ER. B) Intracellular GFP localization in the indicated *S. cerevisiae* strains after overnight growth minimal glucose medium. Scale bar represents 5 µm. C) Immunoblot analysis of (SP)-G-eGFP-LPETG-HDEL-Myc substrate modification by soluble ER-SrtA and TM_Sec__66_-SrtA under control of the *GAL* promoter. Samples were collected at 0, 4 and 8 h of galactose induction. Total cell pellet lysates were analyzed (top) and anti-GFP immunoprecipitation was performed on the culture media to detect secreted GFP (bottom). Fractions marked with asterisks were analyzed by MS. D) MS/MS spectrum of a tryptic fragment of the circular GFP showing the ligation of C-terminal residues (LPET) to the N-terminal residues (GTIPLGSVSK). The linkage peptide was found in the (**) secreted GFP samples, but not in the (*) pellet or secreted samples. E) Quantification of secreted circular GFP product. A standard 50 mL galactose induction starting with 10 OD units (P, 0 h, ∼350 µg of total protein) yielded about 0.75 µg of circular GFP product (S, 8 h) as compared to purified GFP. Experiments were performed multiple times, representative experiments are shown.

Desirable properties of circular proteins include increased stability and biological activity (10—12) and presumably increased resistance to exoproteolytic attack as well. Current sortase-mediated methods for the production of circular peptides and proteins involve peptide synthesis or bacterial expression, followed by *in vitro* sortase reactions and subsequent purification of the desired reaction product. We addressed the production of circular peptides using intracellular sortagging in the ER of *S. cerevisiae*. A GFP ER circularization substrate was designed that contained the Pho5 signal peptide and a C-terminal HDEL sequence for ER retention, (SP)-G-GFP-LPETG-Myc-HDEL. The Pho5 signal peptide was chosen because signal peptide cleavage in this case is predicted to expose an N-terminal glycine 2011 as the necessary nucleophile. Successful circularization of this GFP substrate should result in loss of the HDEL ER retention signal and subsequent release of the circular product from the ER (Figure 3A). The (SP)-G-GFP-LPETG-Myc-HDEL substrate was expressed under control of the constitutive CIT1 promoter and showed a clear cortical ER localization after overnight growth compared to cytosolic localization of the G-GFP-LPETG-Myc substrate described above (Figure 3B). A soluble ER-SrtA was generated through addition of the Pho5 signal peptide and HDEL sequences (Sol. ER-SrtA); a membrane-anchored SrtA was designed by fusion to the *S. cerevisiae* Sec66 transmembrane fragment (TM_Sec66_-SrtA), predicted to yield a membrane-anchored sortase variant with its active site exposed to the ER lumen. Both sortase constructs were placed under control of the inducible GAL promoter.

Galactose induction was performed on *S. cerevisiae* strains expressing the ER GFP substrate without sortase or either of the two ER sortase variants. To analyze the different GFP species formed during the intracellular sortase reaction, cell pellets were harvested after 0, 4 and 8 h of induction and prepared for immunoblot analysis. In parallel, the presence of secreted GFP in the culture supernatant was analyzed by anti-GFP immunoprecipitation. After 4 and 8 h of galactose induction, we observed high and moderate expression of the soluble ER-SrtA and TM_Sec66_-SrtA, respectively. Soluble ER-SrtA and TM_Sec66_-SrtA partially converted the GFP substrate, as indicated by the disappearance of the slowest migrating GFP species and loss of the C-terminal Myc tag over time (Figure 3C, top). Even though expression of TM_Sec66_-SrtA was lower than expression of ER-SrtA, the former consistently showed a faster conversion rate of the GFP substrate at 4 h after galactose induction. A pattern of multiple faint and faster migrating GFP polypeptides was detected in the pellet fraction. The secreted GFP fractions showed a faster migrating GFP band in those strains expressing ER-SrtA or TM_Sec66_-SrtA after 8 h of galactose induction (Figure 3C, bottom). MS analysis of the 8-h cell pellet and culture supernatant samples (*) revealed that only the culture supernatant samples from the soluble ER-SrtA and TM_Sec66_-SrtA contained the circular GFP peptide (**) (Figure 3D). A standard galactose induction experiment starting with 10 optical density (OD) units of cells (∼350 µg of total protein) in 50 mL media yielded about 0.75 µg of secreted circular peptide (Figure 3E). The MS and quantification results clearly demonstrate that secretion of the circular GFP without the HDEL sequence is very efficient. These experiments show that ER sortagging allows the production of secreted circular polypeptides with the possibility of protein modifications specific to eukaryotes (e.g. N-linked glycosylation).

### Kinetics of sortase reactions with Rac1 substrate and G_5_-eGFP nucleophile

**Figure 4:**
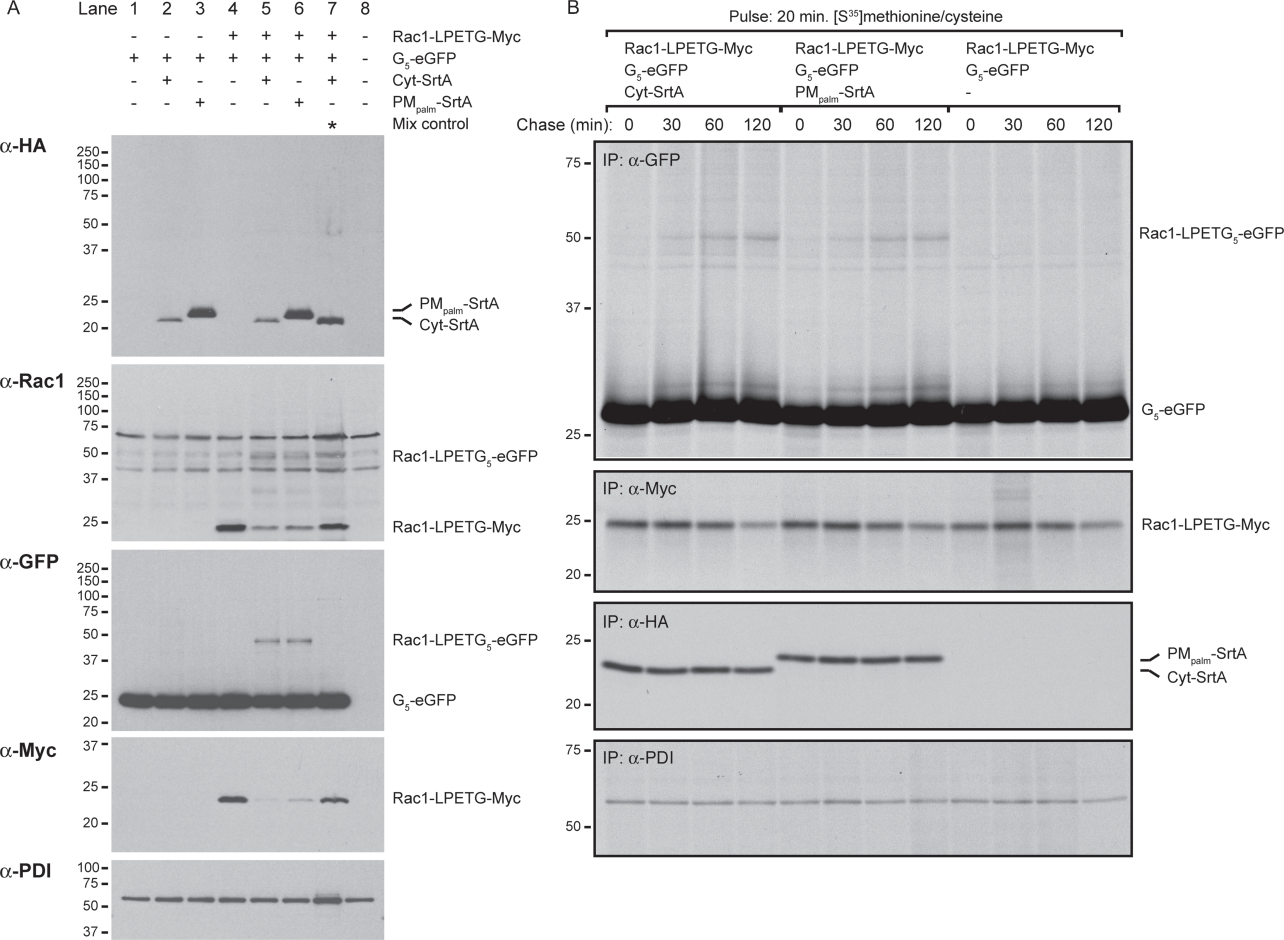
Kinetics of cytosolic sortase reactions with Rac1 substrate and G_5_-eGFP nucleophile A) Immunoblot analysis of HEK293T cells transfected with different combinations of Rac1-LPETG-Myc substrate, G_5_-eGFP nucleophile and Cyt-SrtA or PM_palm_-SrtA plasmids. Cells expressing Rac1 substrate and G_5_-eGFP nucleophile were mixed with cells expressing Cyt-SrtA before lysis and treated like the other samples (mix control). B) Radioactive pulse-chase experiment on HEK293T cells transfected with the indicated Rac1 substrate, GFP nucleophile and sortase constructs. Cells were pulse-labeled for 20 min with [^35^S]methionine/cysteine and chased for 0, 30, 60 and 120 min. Immunoprecipitations (IP) were performed with anti-GFP, anti-Myc, anti-HA or anti-PDI antibodies. All experiments were performed multiple times, representative experiments are shown.

To investigate the efficiency of the sortase reaction in the mammalian cytosol in more detail, we chose the small GTPase Rac1 as a substrate. We replaced the C-terminal CaaX-box motif of mouse Rac1 with a sortase tag and a Myc tag, resulting in a Rac1-LPETG-Myc substrate. The glycine at position 12 was changed to valine to yield a constitutively active Rac1_G12V_ variant 1995. The Rac1-LPETG-Myc substrate was expressed in HEK293T cells in combination with a cytosolic GFP-based nucleophile (G_5_-eGFP) and Cyt-SrtA or PM_palm_-SrtA. Co-expression of the Rac1-LPETG-Myc substrate and the G_5_-eGFP nucleophile with either sortase construct resulted in an almost complete loss of the Myc tag and the appearance of the Rac1-LPETG_5_-eGFP reaction product (Figure 4A). In contrast to some of the other experiments described in this paper, almost no acyl-enzyme intermediates were detected (Figure 4A, top), suggesting that the SrtA-Rac1 intermediate is quickly resolved. To study the kinetics of the Rac1 sortagging reaction, we performed a pulse-chase experiment. HEK293T cells expressing the Rac1-LPETG-Myc substrate, G_5_-eGFP nucleophile and Cyt-SrtA, PM_palm_-SrtA or no sortase were pulse-labeled with [S^35^]methionine/cysteine for 20 min and chased for the indicated times to follow the population of labeled proteins over time. Cell lysates were used for immunoprecipitation with anti-GFP antibody to follow formation of Rac1-LPETG_5_-eGFP over time. Additional immunoprecipitations were performed with anti-Myc, anti-HA and anti-PDI antibodies. The Rac1-LPETG_5_-eGFP reaction product was detectable in both sortase-expressing conditions after 30 min of chase, showing that the sortase reaction occurs rapidly after protein translation (Figure 4B). The Rac1-LPETG_5_-eGFP ligation product accumulated equally in the Cyt-SrtA and PM_palm_-SrtA conditions. Anti-Myc immunoprecipitation showed a gradual disappearance of the Myc tag during the chase, independent of sortase expression, indicating that this is most likely due to normal degradation of the Rac1-LPETG-Myc substrate. These experiments demonstrate that ligation products can be formed between a protein substrate (Rac1) and an unrelated protein nucleophile (GFP) in the cytosol.

### Efficiency of ER luminal Kar2 sortagging is determined by nucleophile and sortase type

**Figure 5:**
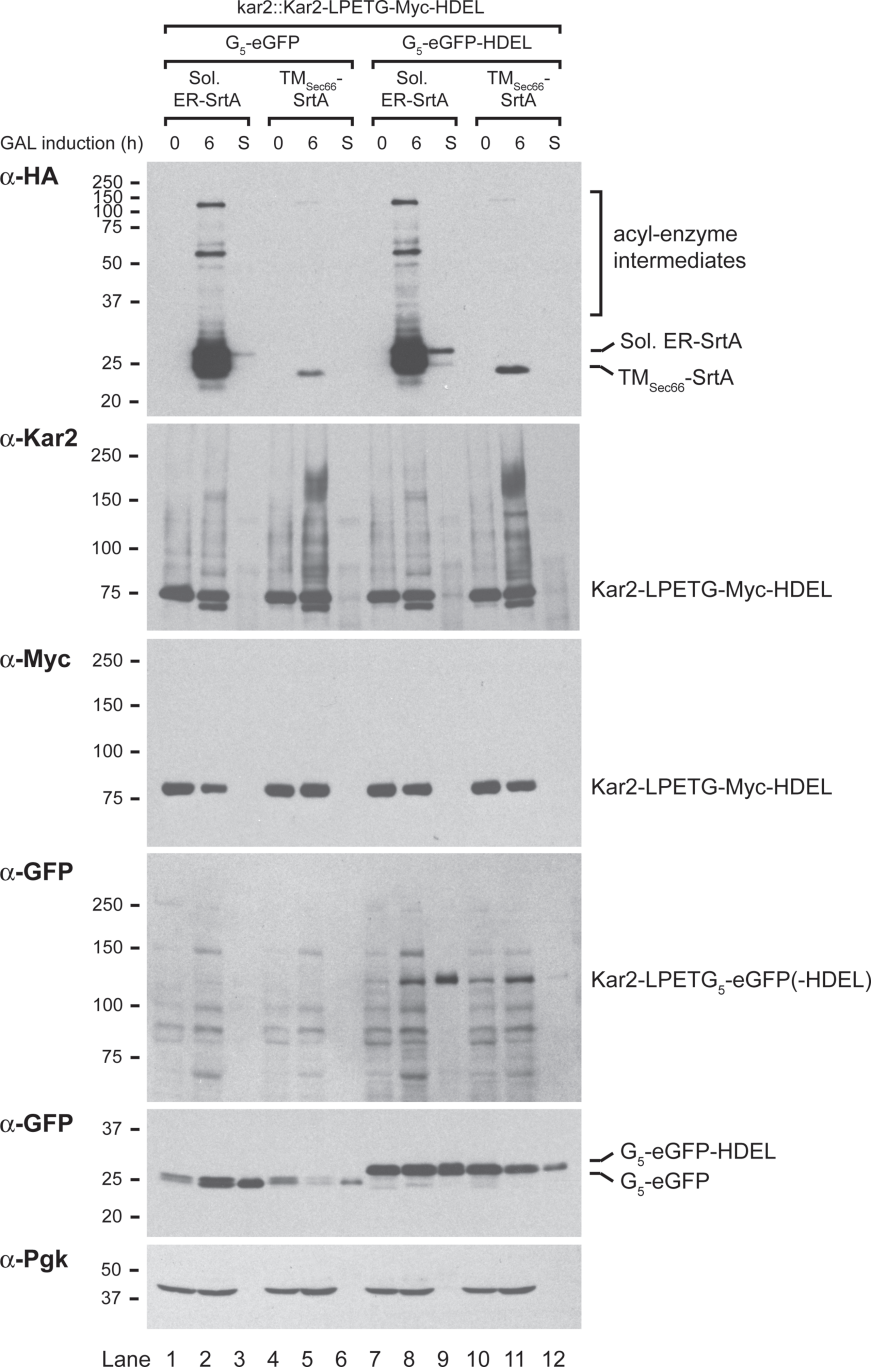
ESortagging of Kar2 substrate with G_5_-GFP nucleophiles in the ER lumen Galactose induction of *S. cerevisiae* kar2::Kar2-LPETG-Myc-HDEL strain constitutively expressing (SP)-G_5_-eGFP or (SP)-G_5_-eGFP-HDEL nucleophile with soluble ER-SrtA or TM_Sec__66_-SrtA under control of the GAL promoter. Sortase expression was induced by growth on galactose-containing media. Cell pellets were harvested (0 and 6 h fractions) and an anti-GFP immunoprecipitation was performed on culture media of the 6-h time-point to detect secreted GFP (S fractions). The experiment was performed multiple times, a representative experiment is shown.

Our data show that sortase can be used for intermolecular protein ligation in the cytosol. Does a similar approach work for sortagging in the ER lumen? We chose the highly expressed yeast ER luminal chaperone Kar2 (the ortholog to mammalian BiP) as a substrate to explore this question. A *S. cerevisiae* strain was generated in which the genomic copy of the *KAR2* gene was replaced by a gene encoding Kar2-LPETG-Myc-HDEL. We transformed this kar2::Kar2-LPETG-Myc-HDEL strain with two different GFP-based nucleophiles: (SP)-G_5_-eGFP and (SP)-G_5_-eGFP-HDEL. Both resulting strains were then transformed with the galactose-inducible soluble ER-SrtA or the TM_Sec66_-SrtA constructs, resulting in four strains with different combinations of nucleophiles and sortases. Galactose induction was performed on the four strains and cell pellets and culture supernatants were harvested at the indicated time-points. Anti-GFP immunoprecipitation was performed on the culture supernatants to detect secreted GFP products. After 6 h of galactose induction, we observed high and moderate expressions of the soluble ER-SrtA and TM_Sec66_-SrtA, respectively. These expression levels were consistent with the ER GFP circularization experiment (see Figure 3). The (SP)-G_5_-eGFP and (SP)-G_5_-eGFP-HDEL nucleophiles were detectable both in the pellet and in the secreted fractions, but a higher percentage of the G_5_-eGFP-HDEL nucleophile was retained in the cell pellet compared to the G_5_-eGFP nucleophile. We hypothesize that secretion of the HDEL-containing nucleophile is due to overloading of the HDEL receptor, as also the soluble ER-SrtA construct contains an HDEL retention signal. Multiple modifications of the Kar2-LPETG-Myc-HDEL substrate were detectable at the 6-h galactose induction time-point (Figure 5). First, a distinct and faster migrating Kar2-reactive product was detected, most likely due to hydrolysis of the acyl-enzyme intermediate, as the product did not contain the Myc tag. Second, multiple slower migrating Kar2-reactive polypeptides were generated following induction of sortase. Interestingly, induction of ER-SrtA or TM_Sec66_-SrtA led to the formation of different products, presumably because of different localization of the two SrtA constructs within the ER and the nucleophile proximity in their respective locations. Third, a GFP-reactive band of around the expected molecular weight of the Kar2-LPETG_5_-eGFP product was detected. The most pronounced Kar2-LPETG_5_-eGFP product was reproducibly found in the supernatant of the strain expressing ER-SrtA and the (SP)-G_5_-eGFP-HDEL nucleophile (Figure 5, lane 9). This experiment shows that sortase can be employed for intermolecular ligation in the ER lumen and that the efficiency of the reaction is determined by the combination of the appropriate substrate, nucleophile and sortase.

### Intermolecular protein ligation using p97 substrate and G_5_-eGFP nucleophile

The hexameric AAA-ATPase p97 (also known as valosin-containing protein in mammals and Cdc48 in *S. cerevisiae*) is a key player in 26S proteasome-dependent protein degradation, as well as in many other cellular processes. *In vitro* sortase-mediated crosslinking of p97 monomers results in the formation of p97 oligomers 2009. The G-His-p97-LPSTG substrate used in this study contains a C-terminal sortase-recognition motif and an N-terminal glycine. Incubation of this p97 substrate with *S. aureus* SrtA results in a chain of C- to N-terminally linked monomers that can be resolved by incubation with diglycine in the *in vitro* reaction 2009. With the possibility of intracellular sortagging, we set out to investigate the structural properties of the G-His-p97-LPSTG substrate *in vivo*.

**Figure 6:**
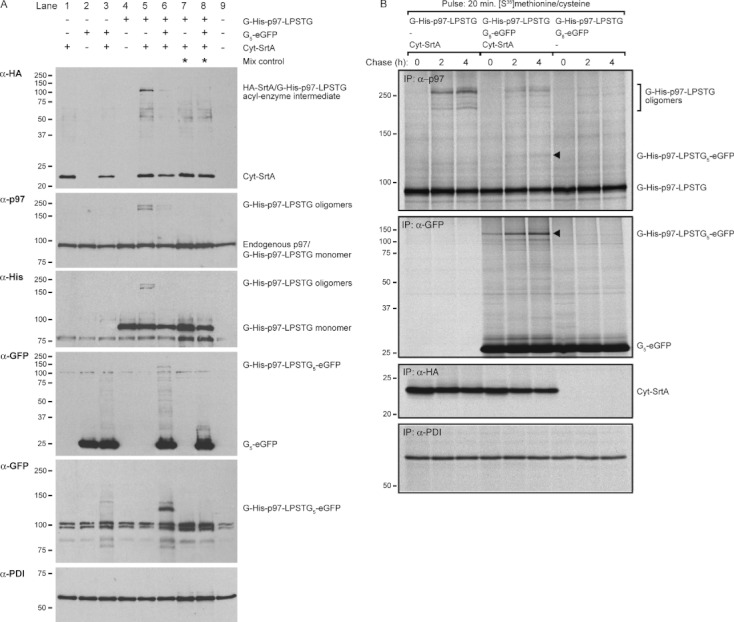
Sortase-mediated protein ligations using p97 substrate and G_5_-eGFP nucleophile A) Immunoblot analysis of HEK293T cells expressing different combinations of the G-His-p97-LPSTG substrate, G_5_-eGFP nucleophile and Cyt-SrtA constructs. Cells expressing either substrate(s) or Cyt-SrtA were mixed before lysis and treated like the other samples (mix control). B) Radioactive pulse-chase experiment on HEK293T cells transfected with the indicated p97 substrate, GFP nucleophile and sortase constructs. Cells were pulse-labeled for 20 min with [^35^S]methionine/cysteine and chased for 0, 2 and 4 h. Immunoprecipitations (IP) were performed with anti-p97, anti-GFP, anti-HA or anti-PDI antibodies. Arrows indicate the p97-GFP ligation product. Experiments were performed multiple times, representative experiments are shown.

HEK293T cells were transfected with different combinations of the p97 substrate, Cyt-SrtA and the cytosolic G_5_-eGFP nucleophile. Co-expression of G-His-p97-LPSTG with sortase resulted in the formation of multiple more slowly migrating polypeptides with a molecular weight of >190 kDa (Figure 6A, lane 5), absent from the corresponding mix control (Figure 6A, lane 7). The molecular weights of these materials do not obviously correspond to the linear p97 oligomers observed in the *in vitro* experiment 2009 and we did not detect a C- to N-terminal fused p97 product by MS (data not shown). Therefore, these higher molecular weight products may represent non-linear reaction products, possibly involving the use of ε-amines of lysine residues within the p97 polypeptide, in close proximity to the covalent acyl-enzyme intermediates, as the incoming nucleophiles (4, 21). Within the intracellular hexameric complex, the p97 N-terminus may not be available for sortagging, for example, because of associated interaction partners absent from recombinant p97 exposed to sortase *in vitro*. Co-expression of the G_5_-eGFP nucleophile resulted in a reduction of the p97 oligomers and the appearance of a p97-GFP fusion product (marked G-His-p97-LPSTG_5_-eGFP) (Figure 6A, lane 6) that was absent from the corresponding mix control (Figure 6A, lane 8). To investigate the kinetics and efficiency of the p97 sortagging reactions, we performed a pulse-chase experiment. The p97 oligomers were readily detectable in the presence of Cyt-SrtA and consisted of <10% of total p97 present in the pulse (Figure 6B, top). The p97-GFP fusion product was only a minor fraction of the total p97 present in the pulse, but was clearly detectable in the GFP immunoprecipitation (Figure 6B, arrows). We conclude that sortase can be employed for ligation reactions within a protein complex as well as for intermolecular ligations between a protein substrate (p97) and an unrelated protein nucleophile (GFP).

## Discussion

Here, we show that the Ca^2+^-independent *S. pyogenes* SrtA enzyme can be used for site-specific protein–protein ligation reactions in different intracellular compartments. Various protein substrates, when equipped with the small five amino acid C-terminal sortag (LPXTG) and/or a N-terminal (oligo)glycine, allowed successful inter- and intramolecular protein ligations in the cytosol and ER lumen of *S. cerevisiae* and HEK293T cells.

We show that performing the sortase reaction on a GFP circularization substrate in the ER lumen leads to secretion of the circular product (Figure 3). Sortase-mediated circularization of four-helix bundle cytokines (such as IFNα3 and GCSF-3), human EPO and the wound-healing peptide histatin-1 increased their stability and biological activity compared to their linear counterparts (10—12). ER luminal production of circular polypeptides opens up the possibility to generate natively folded recombinant proteins with protein modifications specific for eukaryotes, including glycosylation and other complex sugar additions.

In addition to intramolecular protein ligations such as circularization of the GFP substrate, we show that Rac1, Kar2 and p97 substrates can all be sortagged with the appropriate GFP-based nucleophiles (Figures 6). These experiments demonstrate that protein ligations can be accomplished between a substrate and a soluble unrelated nucleophile. In addition, intracellular sortagging can be employed to investigate proximity relationships in protein complexes, as demonstrated by the formation of higher order structures between p97 monomers within the p97 hexamer (Figure 6). As possible applications of sortase-mediated intracellular protein modification, we envision the possibility of further exploring proximity relationships within protein complexes and changes therein, or of otherwise altering properties of the newly formed protein–protein adducts, such as changes in stability or intracellular localization. Another application of the sortase method could be the site-specific controlled attachment of a protein such as ubiquitin. In conclusion, the intracellular labeling method enables site-specific tagging under control of a constitutive or inducible promoter, leading to rapid formation of reaction products at the location of choice.

Controlling the intracellular sortagging reaction may be complicated by possible reactions that involve ε-amino groups of lysines that can act as nucleophiles (4, 21) if in close proximity of the thioacyl bond, as well as hydrolysis of the acyl-enzyme intermediate 2000. The oligomers in the p97 experiment (Figure 6) may be the reaction products involving lysine nucleophiles. Loss of the Myc tag in the Rac1 and Kar2 experiments is most likely (partially) due to hydrolysis (Figures 4 and 5). An interesting observation is the variation in levels of acyl-enzyme intermediates detectable in the different experiments. While almost no acyl-enzyme intermediates were seen in the *S. cerevisiae* GFP circularization or Rac1 experiments (Figures 1 and 2), acyl-enzyme intermediates were clearly detectable in the HEK293T GFP circularization, Kar2 and p97 experiments (Figures 1, 5 and 6). We hypothesize that nucleophile availability largely accounts for this difference and that resolving the acyl-enzyme intermediate is consequently the limiting step of the intracellular sortase reaction. The expression of adequate levels or an increase in the local concentration of nucleophile is most likely to increase product yields. We attempted to increase the product yield of the Rac1-LPETG-Myc to G_5_-eGFP ligation by the introduction of β-hairpin domains in both the substrate and nucleophile. Yamamura et al. 2011 showed that introduction of a rigid β-hairpin structure around the ligation site increased *in vitro* reaction yields with *S. aureus* SrtA. However, intracellular ligation of the Rac1 and GFP hairpin constructs using *S. pyogenes* SrtA was unsuccessful (data not shown). Both differences in enzyme specificity or intracellular reaction conditions may account for this difference between *in vitro* and *in vivo* ligation efficiency. Other means of increasing intracellular sortagging yields need to be explored. A key challenge that remains is the intracellular delivery of small molecules at sufficient levels to expand the range of possible nucleophiles for the intracellular sortagging method. The design of such cell-permeant probes would enhance further the utility of this protein ligation method.

## Materials and Methods

### Expression plasmids

Sortase, substrate and nucleophile expression plasmids for *S. cerevisiae* and mammalian cell are listed in Table 1. For expression of proteins in *S. cerevisiae*, genes were cloned into integration plasmids pRS303 (containing the HIS3 selection marker) or pRS306 (containing the URA3 selection marker) 1989. For inducible and constitutive expression, we cloned either the *GAL* promoter (pGAL) or the 600-bp upstream region of the citrate synthase gene (pCIT1), respectively, into the pRS303 and pRS306 plasmids. For protein expression in mammalian cells, all genes were cloned into the *CMV* promoter-containing plasmid pcDNA3.1(-) (Invitrogen). SignalP 4.0 was used for the prediction of signal peptide cleavage sites 2011. Full amino acid sequences of all engineered proteins used in this study are listed in Figure S1.

**Table 1:** Sortase, substrate and nucleophile constructs used in this study

	Cell type	Plasmid	Nucleophile constructs
*S. aureus* SrtA	*S. cerevisiae*	pRS303-pGAL-HA-SrtA_staph_	pKS115
*S. pyogenes* SrtA	S. cerevisiae	pRS303-pGAL-HA-SrtA_strep_	pKS78
Cyt-SrtA	HEK293T	pcDNA3.1(-)-HA-SrtA_strep_	pKS77
PM_palm_-SrtA	HEK293T	pcDNA3.1(-)-PM-C_palm_-HA-SrtA	pKS37
Sol. ER-SrtA	*S. cerevisiae*	pRS303-pGAL-SP_PHO5_-G-SrtA_strep_-HA-HDEL	pKS82
TM_Sec66_-SrtA	*S. cerevisiae*	pRS303-pGAL-HA-TM_Sec66_-SrtA	pKS105
G-eGFP-LPETG-Myc	*S. cerevisiae*	pRS306-pGAL-G-eGFP-LPETG-Myc	pKS85
G-eGFP-LPETG-Myc	HEK293T	pcDNA3.1(-)-G-eGFP-LPETG-Myc	pKS93
(SP)-G-eGFP-LPETG-Myc-HDEL	S. cerevisiae	pRS306-pCIT1-SP_PHO5_-G-eGFP-LPETG-Myc-HDEL	pKS71
Kar2-LPETG-Myc-HDEL	*S. cerevisiae*	pRS306-pCIT1-Kar2-LPETG-Myc-HDEL	pKS116
G-His-p97-LPSTG	HEK293T	pcDNA3.1(-)-G-His6-p97-LPSTG-SGGG	pKS36
Rac1-LPETG-Myc	HEK293T	pcDNA3.1(-)-Rac1_G12V_-LPETG-Myc	pKS108
G_5_-eGFP	HEK293T	pcDNA3.1(-)-G_5_-eGFP	pKS110
(SP)-G_5_-eGFP	*S. cerevisiae*	pRS306-pCIT1-SP_PHO5_-G_5_-eGFP	pKS90
(SP)-G_5_-eGFP-HDEL	*S. cerevisiae*	pRS306-pCIT1-SP_PHO5_-G_5_-eGFP-HDEL	pKS103

### Yeast strains and culture conditions

For routine non-selective culturing of *S. cerevisiae*, strains were grown in YPDA (2% bactopeptone, 1% yeast extract, 2% glucose and 5.5 µg/mL adenine) at 30°C. *Saccharomyces cerevisiae* genomic integration strains were generated by transformation of wild-type strain W303 with linearized pRS303 or pRS306-based plasmids and selection with the appropriate marker. Transformants were selected on solid minimal medium containing 0.67% yeast nitrogen base (YNB) w/o amino acids (DIFCO), 2% glucose, 2% agar and amino acids as needed (5.5 µg/mL adenine, 20.9 µg/mL histidine, 2.2 µg/mL uracil and 13.1 µg/mL leucine). A kar2::Kar2-LPETG-Myc-HDEL genomic replacement strain was generated by transformation with a polymerase chain reaction (PCR) product that contained the engineered Kar2-LPETG-Myc-HDEL gene with 5^′^ and 3^′^ flanking Kar2 regions for homologous recombination. Integration of the PCR product in the correct genomic locus was confirmed by PCR analysis. For galactose induction, cells were pregrown in media containing YNB with 2% glucose and amino acids as needed, grown overnight and transferred to media containing YNB and 0.3% glucose with amino acids and grown for 16 h. The next morning, cells were inoculated in YPGA (2% bactopeptone, 1% yeast extract, 2% galactose and adenine) to an OD at 600 nm of 0.2. Samples were taken at the indicated time-points after galactose induction.

### Mammalian cell lines and culture conditions

Mammalian kidney-derived HEK293T cells were purchased from ATCC and grown in DMEM media with 10% inactivated fetal bovine serum. HEK293T cells were transfected with pcDNA3.1-based expression plasmids using TransIT transfection reagent (Mirus) according to the manufacturer's instructions. Cells were harvested 18–24 h after transfection.

### Immunoprecipitation, blotting and antibodies

*Saccharomyces cerevisiae* cell lysates were prepared by glass bead lysis in PBS with protease inhibitors and phenylmethylsulfonyl fluoride on a vortex at 4°C. HEK293T cells were lysed in 1% SDS in PBS. For immunoprecipitation of secreted GFP products, *S. cerevisiae* culture medium was harvested and incubated with anti-GFP antibody (Abcam) and Protein-A beads (RepliGen). Anti-HA-fluorescein (Roche) was used for immunofluorescence on HEK293T cells. For immunoblotting, protein extracts were separated on an 8, 10 or 12% SDS–polyacrylamide gel and blotted to nitrocellulose membrane using a semi-dry system. Antibodies used were anti-HA- horseradish peroxidase (HRP) (Roche), anti-Myc-HRP (Cell Signaling), anti-His-HRP (Qiagen), anti-GFP-HRP (Santa Cruz), anti-ScKar2 (a kind gift of Dr Matthias Seedorf), anti-p97 (Ploegh lab), anti-Rac1 (Millipore), anti-ScPgk (Molecular Probes) and anti-PDI (Ploegh lab) in combination with goat anti-mouse or goat anti-rabbit HRP-conjugated secondary antibodies (SouthernBiotech) when necessary.

### Immunofluorescence and microscopy

*Saccharomyces cerevisiae* strains were grown as indicated, harvested by centrifugation, fixed for 20 min in 4% paraformaldehyde (PFA) and prepared for microscopy. For immunofluorescence, HEK293T cells were prepared by growth on coverslips and transfected 24 h prior the experiment. Cells were fixed in 4% PFA for 20 min and quenched for 10 min in 20 mm glycine and 50 mm NH_4_Cl/PBS. Permeabilization was performed in 0.1% Triton-X-100/PBS for 10 min and blocking by addition of 4% BSA/PBS for 10 min. Fluorescein-conjugated anti-HA antibody (Roche) was added at a 1:100 dilution in 2% BSA/PBS for 45 min in a humidified chamber. Cells were prepared for microscopy by mounting on slides in Fluoromount-G mounting media (SouthernBiotech). Images were collected with a Perkin-Elmer spinning disk confocal microscope with 100× magnification using Volocity software.

### Pulse-chase experiments

For the Rac1 experiments, HEK293T cells were grown in six-well plates and transfected with the appropriate plasmids. The next day, cells were incubated with methionine- and cysteine-free DMEM for 30 min at 37°C. Cells were labeled with 170 µCi [^35^S]methionine/cysteine (1175 Ci/mmol; PerkinElmer Life Sciences) per well at 37°C for 20 min and chased with DMEM supplemented with nonradiolabeled methionine (2.5 mm) and cysteine (0.5 mm) at 37°C for the indicated times. For the p97 experiments, HEK293T cells were grown and transfected in 10-cm dishes and the pulse was performed on cells in suspension. Cells were lysed in 150 µL of 1% SDS in PBS after which 1500 µL of Nonidet P-40 (NP-40) buffer was added (25 mm Tris pH 7.4, 5 mm MgCl_2_, 150 mm NaCl and 0.5% NP-40). Immunoprecipitations were performed using Protein-A beads (IPA 300, RepliGen) or Protein-G beads (Sigma) and the relevant antibody for at least 3 h at 4°C with gentle agitation. Immune complexes were eluted by boiling in reducing sample buffer and subjected to SDS–PAGE analysis (12%). Gels were dehydrated in dimethylsulfoxide (DMSO), treated with 2,5-diphenyloxazole in DMSO and visualized by autoradiography 1974.

### Mass spectrometry

For MS analysis of the circular GFP, immunoprecipitation was performed with anti-GFP antibody (Abcam) and Protein-A beads (RepliGen). G-His-P97-LPSTG crosslinking products were concentrated using NiNTA agarose (Qiagen). Eluates were run on SDS–PAGE gels and stained with Coomassie. Appropriate areas were cut from the gel and gel fragments were subjected to trypsin and/or chymotrypsin digestion. The resulting peptides were extracted and separated by reversed-phase high-performance liquid chromatography and followed by MS/MS analysis. The mass spectral data were database searched using sequest.

## Supporting Information

Additional Supporting Information may be found in the online version of this article: **Figure S1:** Protein sequences of sortase, substrate and nucleophile constructs used in this study. Please note: Wiley-Blackwell are not responsible for the content or functionality of any supporting materials supplied by the authors. Any queries (other than missing material) should be directed to the corresponding author for the article.
