# Supplementary material for: Protein Ligation in Living Cells Using Sortase
Source: Traffic. 2012 Mar 23;13(6):780–9. doi: 10.1111/j.1600-0854.2012.01345.x (PMC3490390; doi:10.1111/j.1600-0854.2012.01345.x)
Supplement: Supplementary file 1 — Additional Supporting Information may be found in the online version of this article: Figure S1: Protein sequences of sortase, substrate and nucleophile constructs used in this study. Please note: Wiley-Blackwell are not responsible for the content or functionality of any supporting materials supplied by the authors. Any queries (other than missing material) should be directed to the corresponding author for the article. [file tra0013-0780-SD1.doc]

Supplementary Information: Protein sequences of sortase, substrate and nucleophile constructs used in this study.

>HA-SrtAstaph (S. aureus)
MRSSYPYDVPDYASSGLVPRGSHMQAKPQIPKDKSKVAGYIEIPDADIKE
PVYPGPATPEQLNRGVSFAEENESLDDQNISIAGHTFIDRPNYQFTNLKA
AKKGSMVYFKVGNETRKYKMTSIRDVKPTDVGVLDEQKGKDKQLTLITCD
DYNEKTGVWEKRKIFVATEVK*

>HA-SrtAstrep (S. pyogenes)
MRSSYPYDVPDYASSGLVPRGSVLQAQMAAQQLPVIGGIAIPELGINLPI
FKGLGNTELIYGAGTMKEEQVMGGENNYSLASHHIFGITGSSQMLFSPLE
RAQNGMSIYLTDKEKIYEYIIKDVFTVAPERVDVIDDTAGLKEVTLVTCT
DIEATERIIVKGELKTEYDFDKAPADVLKAFNHSYNQVST*

>Cyt-SrtA
MRSSYPYDVPDYASSGLVPRGSVLQAQMAAQQLPVIGGIAIPELGINLPI
FKGLGNTELIYGAGTMKEEQVMGGENNYSLASHHIFGITGSSQMLFSPLE
RAQNGMSIYLTDKEKIYEYIIKDVFTVAPERVDVIDDTAGLKEVTLVTCT
DIEATERIIVKGELKTEYDFDKAPADVLKAFNHSYNQVST*

>PMpalm-HA-SrtA
MGCIKSKGKDSAGAIDAYPYDVPDYAHSAGGSVLQAQMAAQQLPVIGGIA
IPELGINLPIFKGLGNTELIYGAGTMKEEQVMGGENNYSLASHHIFGITG
SSQMLFSPLERAQNGMSIYLTDKEKIYEYIIKDVFTVAPERVDVIDDTAG
LKEVTLVTCTDIEATERIIVKGELKTEYDFDKAPADVLKAFNHSYNQVST
*

>Sol. ER-SrtA 
MFKSVVYSILAASLANAGTIPLGSSVLQAQMAAQQLPVIGGIAIPELGIN
LPIFKGLGNTELIYGAGTMKEEQVMGGENNYSLASHHIFGITGSSQMLFS
PLERAQNGMSIYLTDKEKIYEYIIKDVFTVAPERVDVIDDTAGLKEVTLV
TCTDIEATERIIVKGELKTEYDFDKAPADVLKAFNHSYNQVSTVDAAAYP
YDVPDYANGAASEHDEL*

>TMSec66-SrtA
MYPYDVPDYAETKSISVYTPLIYVFILVVSLVMFASSGSEPVSTESVLQA
QMAAQQLPVIGGIAIPELGINLPIFKGLGNTELIYGAGTMKEEQVMGGEN
NYSLASHHIFGITGSSQMLFSPLERAQNGMSIYLTDKEKIYEYIIKDVFT
VAPERVDVIDDTAGLKEVTLVTCTDIEATERIIVKGELKTEYDFDKAPAD
VLKAFNHSYNQVST*

>G-eGFP-LPETG-Myc
MGTIPLGSVSKGEELFTGVVPILVELDGDVNGHKFSVSGEGEGDATYGKL
TLKFICTTGKLPVPWPTLVTTLTYGVQCFSRYPDHMKQHDFFKSAMPEGY
VQERTIFFKDDGNYKTRAEVKFEGDTLVNRIELKGIDFKEDGNILGHKLE
YNYNSHNVYIMADKQKNGIKVNFKIRHNIEDGSVQLADHYQQNTPIGDGP
VLLPDNHYLSTQSALSKDPNEKRDHMVLLEFVTAAGITLGMDELYKLPET
GVDAAAEQKLISEEDL*

>(SP)-G-eGFP-LPETG-Myc-HDEL
MFKSVVYSILAASLANAGTIPLGSVSKGEELFTGVVPILVELDGDVNGHK
FSVSGEGEGDATYGKLTLKFICTTGKLPVPWPTLVTTLTYGVQCFSRYPD
HMKQHDFFKSAMPEGYVQERTIFFKDDGNYKTRAEVKFEGDTLVNRIELK
GIDFKEDGNILGHKLEYNYNSHNVYIMADKQKNGIKVNFKIRHNIEDGSV
QLADHYQQNTPIGDGPVLLPDNHYLSTQSALSKDPNEKRDHMVLLEFVTA
AGITLGMDELYKLPETGVDAAAEQKLISEEDLNGAASEHDEL*

>Kar2-LPETG-Myc-HDEL
MFFNRLSAGKLLVPLSVVLYALFVVILPLQNSFHSSNVLVRGADDVENYG
TVIGIDLGTTYSCVAVMKNGKTEILANEQGNRITPSYVAFTDDERLIGDA
AKNQVAANPQNTIFDIKRLIGLKYNDRSVQKDIKHLPFNVVNKDGKPAVE
VSVKGEKKVFTPEEISGMILGKMKQIAEDYLGTKVTHAVVTVPAYFNDAQ
RQATKDAGTIAGLNVLRIVNEPTAAAIAYGLDKSDKEHQIIVYDLGGGTF
DVSLLSIENGVFEVQATSGDTHLGGEDFDYKIVRQLIKAFKKKHGIDVSD
NNKALAKLKREAEKAKRALSSQMSTRIEIDSFVDGIDLSETLTRAKFEEL
NLDLFKKTLKPVEKVLQDSGLEKKDVDDIVLVGGSTRIPKVQQLLESYFD
GKKASKGINPDEAVAYGAAVQAGVLSGEEGVEDIVLLDVNALTLGIETTG
GVMTPLIKRNTAIPTKKSQIFSTAVDNQPTVMIKVYEGERAMSKDNNLLG
KFELTGIPPAPRGVPQIEVTFALDANGILKVSATDKGTGKSESITITNDK
GRLTQEEIDRMVEEAEKFASEDASIKAKVESRNKLENYAHSLKNQVNGDL
GEKLEEEDKETLLDAANDVLEWLDDNFETAIAEDFDEKFESLSKVAYPIT
SKLYGGADGSGAADYDDEDEDDDGDYKLPETGVDAAAEQKLISEEDLNGA
ASEHDEL*

>G-His-P97-LPSTG
MGSSHHHHHHSSGLEVLFQGPHMASGADSKGDDLSTAILKQKNRPNRLIV
DEAINEDNSVVSLSQPKMDELQLFRGDTVLLKGKKRREAVCIVLSDDTCS
DEKIRMNRVVRNNLRVRLGDVISIQPCPDVKYGKRIHVLPIDDTVEGITG
NLFEVYLKPYFLEAYRPIRKGDIFLVRGGMRAVEFKVVETDPSPYCIVAP
DTVIHCEGEPIKREDEEESLNEVGYDDIGGCRKQLAQIKEMVELPLRHPA
LFKAIGVKPPRGILLYGPPGTGKTLIARAVANETGAFFFLINGPEIMSKL
AGESESNLRKAFEEAEKNAPAIIFIDELDAIAPKREKTHGEVERRIVSQL
LTLMDGLKQRAHVIVMAATNRPNSIDPALRRFGRFDREVDIGIPDATGRL
EILQIHTKNMKLADDVDLEQVANETHGHVGADLAALCSEAALQAIRKKMD
LIDLEDETIDAEVMNSLAVTMDDFRWALSQSNPSALRETVVEVPQVTWED
IGGLEDVKRELQELVQYPVEHPDKFLKFGMTPSKGVLFYGPPGCGKTLLA
KAIANECQANFISIKGPELLTMWFGESEANVREIFDKARQAAPCVLFFDE
LDSIAKARGGNIGDGGGAADRVINQILTEMDGMSTKKNVFIIGATNRPDI
IDPAILRPGRLDQLIYIPLPDEKSRVAILKANLRKSPVAKDVDLEFLAKM
TNGFSGADLTEICQRACKLAIRESIESEIRRERERQTNPSAMEVEEDDPV
PEIRRDHFEEAMRFARRSVSDNDIRKYEMFAQTLQQSRGFGSFRFPSGNQ
GGALPSTGSGGG*

>Rac1-LPETG-Myc
MQAIKCVVVGDVAVGKTCLLISYTTNAFPGEYIPTVFDNYSANVMVDGKP
VNLGLWDTAGQEDYDRLRPLSYPQTDVFLICFSLVSPASFENVRAKWYPE
VRHHCPNTPIILVGTKLDLRDDKDTIEKLKEKKLTPITYPQGLAMAKEIG
AVKYLECSALTQRGLKTVFDEAIRAVLCPPPVKKRKRKLPETGVDAAAEQ
KLISEEDL*

>G5-eGFP
MGGGGGTIPLGSVSKGEELFTGVVPILVELDGDVNGHKFSVSGEGEGDAT
YGKLTLKFICTTGKLPVPWPTLVTTLTYGVQCFSRYPDHMKQHDFFKSAM
PEGYVQERTIFFKDDGNYKTRAEVKFEGDTLVNRIELKGIDFKEDGNILG
HKLEYNYNSHNVYIMADKQKNGIKVNFKIRHNIEDGSVQLADHYQQNTPI
GDGPVLLPDNHYLSTQSALSKDPNEKRDHMVLLEFVTAAGITLGMDELYK
*

>(SP)-G5-eGFP
MFKSVVYSILAASLANAGGGGGTIPLGSVSKGEELFTGVVPILVELDGDV
NGHKFSVSGEGEGDATYGKLTLKFICTTGKLPVPWPTLVTTLTYGVQCFS
RYPDHMKQHDFFKSAMPEGYVQERTIFFKDDGNYKTRAEVKFEGDTLVNR
IELKGIDFKEDGNILGHKLEYNYNSHNVYIMADKQKNGIKVNFKIRHNIE
DGSVQLADHYQQNTPIGDGPVLLPDNHYLSTQSALSKDPNEKRDHMVLLE
FVTAAGITLGMDELYK*

>(SP)-G5-eGFP-HDEL
MFKSVVYSILAASLANAGGGGGTIPLGSVSKGEELFTGVVPILVELDGDV
NGHKFSVSGEGEGDATYGKLTLKFICTTGKLPVPWPTLVTTLTYGVQCFS
RYPDHMKQHDFFKSAMPEGYVQERTIFFKDDGNYKTRAEVKFEGDTLVNR
IELKGIDFKEDGNILGHKLEYNYNSHNVYIMADKQKNGIKVNFKIRHNIE
DGSVQLADHYQQNTPIGDGPVLLPDNHYLSTQSALSKDPNEKRDHMVLLE
FVTAAGITLGMDELYKVDAAASEHDEL*
